# QuadraPure-Supported Palladium Nanocatalysts for Microwave-Promoted Suzuki Cross-Coupling Reaction under Aerobic Condition

**DOI:** 10.1155/2014/796196

**Published:** 2014-06-25

**Authors:** Kin Hong Liew, Poh Lee Loh, Joon Ching Juan, Mohd Ambar Yarmo, Rahimi M. Yusop

**Affiliations:** ^1^School of Chemical Sciences and Food Technology, Faculty of Science and Technology, Universiti Kebangsaan Malaysia (UKM), 43600 Bangi, Selangor Darul Ehsan, Malaysia; ^2^Nanotechnology & Catalysis Research Centre (NANOCAT), University of Malaya, 50603 Kuala Lumpur, Malaysia

## Abstract

Cross-linked resin-captured palladium (XL-QPPd) was readily prepared by simple physical adsorption onto the high loading QuadraPure macroporous resin and a subsequent reduction process. To enhance the mechanical stability, entrapped palladium nanocatalysts were cross-linked with succinyl chloride. Both transmission electron microscopy images and X-ray diffraction analysis revealed that the palladium nanoparticles were well dispersed with diameters ranging in 4–10 nm. The catalyst performed good catalytic activity in microwave-promoted Suzuki cross-coupling reactions in water under aerobic condition with mild condition by using various aryl halides and phenylboronic acid. In addition, the catalyst showed an excellent recyclability without significant loss of catalytic activity.

## 1. Introduction

Over the past several decades, palladium is one of the most useful transition metal catalysts in variety of transformations in organic synthesis such as Mizoroki-Heck, Sonogashira coupling, Negishi coupling, Stille, and Suzuki coupling reaction [1–5]. The palladium-catalyzed Suzuki coupling reaction between aryl halides and organoboronic acid is an excellent method for the synthesis of various biaryl compounds, which constitute a wide range of fine chemicals, pharmaceuticals, and advanced materials [6–10]. Despite the great efficiency of homogeneous palladium catalysts in various organic reactions, they have several drawbacks to be resolved. The problems of homogeneous catalysts in recovering and recycling lead to the loss of metal and ligands and incorporation of impurities in the products [11]. For chemical and pharmaceutical industries, this is a task of great economic and environmental issues especially when expensive and/or toxic heavy metal complexes are employed [12]. To overcome these drawbacks, the use of ligand-free heterogeneous palladium catalysts is often desirable because it can be recovered and reused by simple filtration without any loss of catalytic activity [13].

Recently, many recoverable supported palladium catalysts have been reported to catalyze Suzuki coupling reactions in the absence of ligands such as polymers, biomaterials, porous silica, carbon nanotube, polyurea, and natural phosphate [13–19]. However, some supported catalysts allow to be reused as they are heterogeneous catalysts, but often result in significant loss of catalytic activities and the leaching of transition metal during the reaction [20]. The disposal of organic solvent after the Suzuki reaction is also a major environmental problem for pharmaceutical industries. The attention of using water as reaction medium for coupling reactions catalyzed by heterogeneous palladium catalyst was increasing due to the rising concerns for the environment [21]. Water is suitable for Suzuki coupling reaction since it is cheap, nontoxic, and of easy recovery of the products [13].

QuadraPure is attracting considerable attention as an alternative solid support for heterogeneous catalysis as well as for the formation of cross-linked captured palladium catalysts. QuadraPure is a commercial available polystyrene resin, which is widely used as a metal scavenger in variety of organometallic synthesis [22, 23]. This macroporous polystyrene QuadraPure is highly robust and chemically resistant-free flowing and it is compatible with organic, aqueous, protic, and aprotic media. Since QuadraPure is a metal scavenger, it is suitable to be used as a solid support for catalyst to avoid metal leaching problem. In this study, we have prepared a new cross-linked captured palladium (XL-QPPd) catalyst by incorporated Pd(OAc)_2_ in QuadraPure. The new catalyst showed good catalytic activity for microwave-promoted Suzuki coupling reaction to synthesis biaryls using water as a solvent. The microwave heating was applied in the heterogeneous system that can lead to higher reaction rate.

## 2. Experimental Section

### 2.1. Reagents and Materials

The solid support QuadraPure EDA (1.5–2.0 mmol/g loading) and high purity palladium acetate (Pd(OAc)_2_, 99.99%) were obtained from Sigma-Aldrich Co., Inc. Bromobenzene (99%), 4-bromoanisole (≥99%), 1-bromo-4-nitrobenzene (99%), 4-bromobenzotrifluoride (99%), 4-iodoanisole (98%), 4-chloroanisole (99%), chlorobenzene (99.99%), iodobenzene (98%), phenylboronic acid (95%), succinyl chloride (95%), and K_2_CO_3_ (≥99%) were used in their commercially available form. Triethylamine was dried with magnesium sulfate before use. 10% Hydrazine hydrate (80%) solution was prepared by dissolve it in methanol solvent. Other analytical solvents were used as received.

### 2.2. Preparation of Captured Palladium Particles onto QuadraPure Particles (XL-QPPd)

The cross-linked captured palladium (XL-QPPd) was readily prepared by treating a mixture of solid support QuadraPure with palladium acetate in toluene at 80°C for 10 min and then at room temperature for 2 hours to yield brown colored resin-captured. The resulting resin was cross-linked with succinyl chloride and triethylamine in dry DMF solvent. Subsequently, the resin was filtered and then treated with hydrazine hydrate in methanol (10%) at room temperature to give black resin palladium catalyst (1.5 mmol of Pd/g). The color of the resin changed to black because of the trapped palladium nanoparticles, Pd^0^ [24, 25].

### 2.3. General Procedure for Suzuki Coupling Reaction of Aryl Halides Using XL-QPPd as Catalyst

Phenylboronic acid (91 mg, 0.75 mmol, 1.5 equiv.), aryl halides (0.5 mmol), potassium carbonate (101 mg, 0.75 mmol, 1.5 equiv.), water (2 mL), and XL-QPPd resin (16.7 mg, 0.05 mmol, and 5 mol% of aryl halide) were added into a 10 mL vial and heated in a microwave (120°C, 100 w) with magnetic stirring for 10 min. The XL-QPPd resin was recovered by simple filtration with solid phase extraction (SPE) and washed with acetone and dried at room temperature. The reaction mixture was extracted with ethyl acetate (3 × 10 mL). The organic layers were combined and washed with distilled water, dried over MgSO_4_, filtered, and concentrated under reduced pressure. The crude product was further purified by column chromatography on silica gel to yield the final product and characterized by NMR.

### 2.4. Instrumentation

The field emission scanning electron microscope (FESEM) measurements were conducted by using a SUPRA 55VP (CARL ZEISS., Oberkochen, Germany) electron microscopy. The existence of palladium on the surface of XL-QPPd resin was carried out with an energy-dispersive X-ray (EDX) analysis (equipped with the SEM). TEM measurements were performed under vacuum by a CM-12 (PHILIPS) transmission electron microscopy. The XL-QPPd resin was dispersed on acetone solution under ultrasonic vibration for 10 mins and one drop of the suspension evaporated onto a carbon-coated copper grid for TEM measurement. The X-ray powder diffraction (XRD) pattern of the catalyst was taken using a D8-Advance (BRUKER, Germany) X-ray diffractometer system. Diffraction data were recorded using continuous scanning at 8 deg/min, step 0.025. ^1^H and ^13^C NMR data of products were obtained using a JEOL ECP 400 MHz (Superconductor) and BRUKER AVANCE 111 600 MHz spectrometer with CDCl_3_ as the solvent. The amount of Pd was determined by ICP-MS (Optima 2000 DV).

## 3. Results and Discussion

### 3.1. Synthesis and Characterization of Cross-Linked Captured Palladium

The cross-linked captured palladium resin (XL-QPPd) was readily prepared via a 3-step procedure [25, 26]. This was based on physical entrapment by the polymers and also on electronic interactions between the electrons of the aryl rings of polystyrene-based polymers and vacant orbitals onto the catalysts, resulting in stronger connections between the components [27, 28]. Firstly, palladium acetate was immobilized and embedded on the surface of QuadraPure in toluene under the atmosphere condition. Second, the catalyst was cross-linked with succinyl chloride to enhance the chemical and physical stability of the catalyst which mean that the palladium is not easy to leach out from the resin. Lastly, the addition of hydrazine hydrate in methanol act as a reducing agent to reduce Pd^2+^ to Pd^0^.

**Figure 1:**
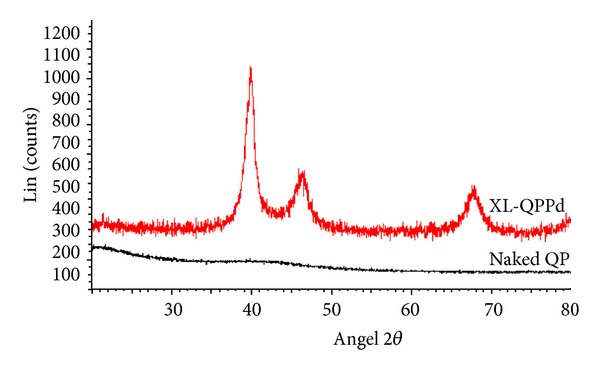
XRD patterns of the naked QuadraPure and the XL-QPPd resin.

The XRD patterns of the naked QuadraPure and the XL-QPPd resin are shown in Figure 1. The result shows the presence of palladium nanoparticles on the QuadraPure surface. After the reduction step, the reflection peaks are observed in XRD pattern at 2*θ* = 40.1°, 46.6°, and 68.3° corresponds to the (111), (200), and (220) planes of fcc (face-centered cubic) lattice, respectively. This data matched the data of Pd^0^ provided by the ASTM (American Society for Testing and Materials) and previous work performed by Shen et al. [29]. The average nanoparticle size is calculated to be 8 nm by using Scherrer formula.

**Figure 2:**
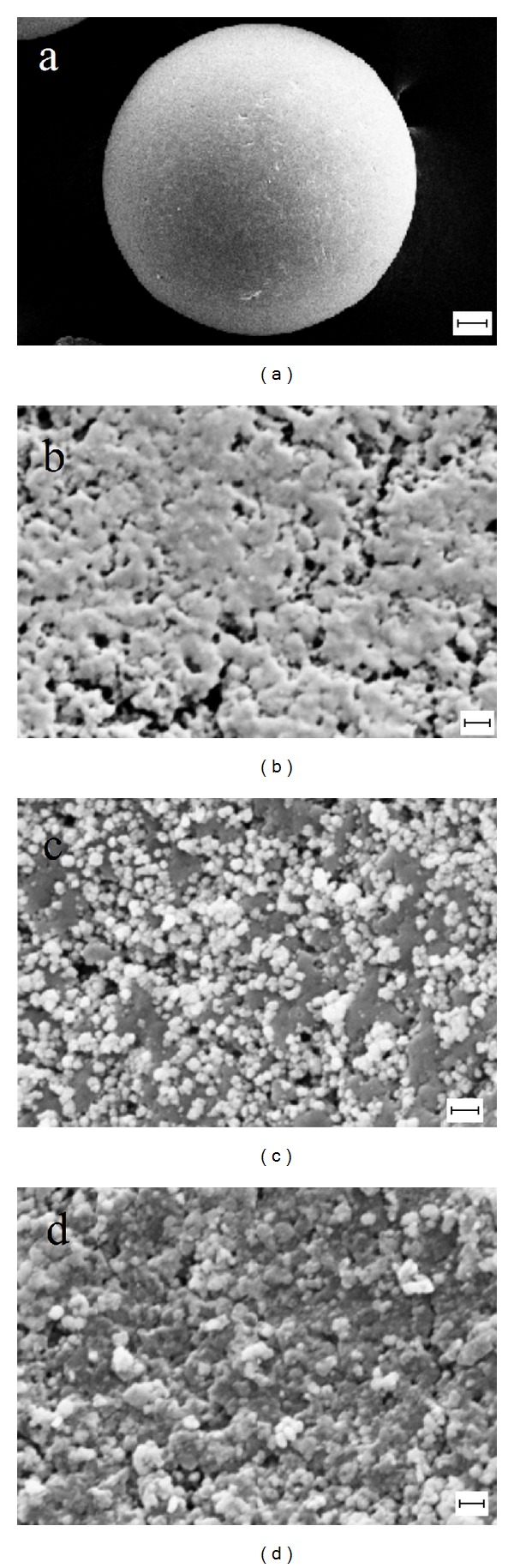
SEM images of (a) and (b) naked QuadraPure (scale bar 20 *μ*m), (c) original XL-QPPd resin and (d) XL-QPPd after ten runs (scale bar: 200 nm).

**Figure 3:**
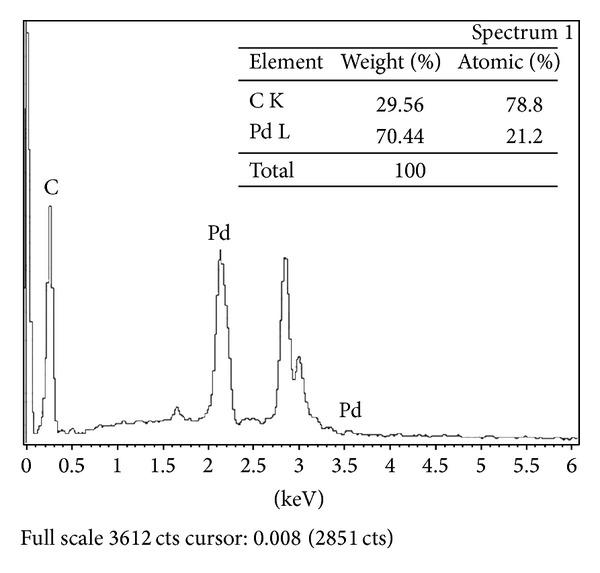
EDX analysis of XL-QPPd resin.

The size and morphology of the supports are revealed by SEM.  Figure 2 shows the SEM images of (a) and (b) naked QuadraPure, (c) original XL-QPPd resin, and (d) XL-QPPd after ten runs. As shown in Figures 2(c) and 2(d), the particles have been formed on the surface of QuadraPure in the entire observed surface, while, for the resin, the clusters are observed. The morphology of XL-QPPd resin after ten runs does not show obvious difference except that the agglomerates become more compact. Scanning electron microscopy with energy dispersive X-ray spectroscopy (SEM-EDX) is the best known and most widely used to determine the elemental composition of a sample. The SEM-EDX analysis of XL-QPPd resin confirmed the immobilization of palladium onto the surface of resin without any impurity (Figure 3).

**Figure 4:**
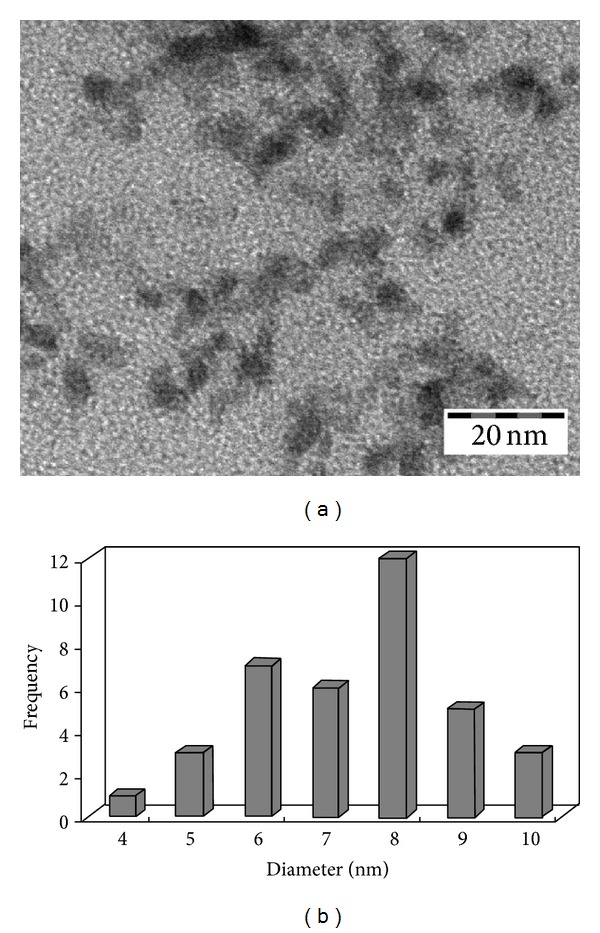
Transmission electron microscopy (TEM) observed physically captured and entrapped on the QuadraPure resin (a) and size distribution of Pd^0^ nanoparticles (b).

The size and distribution of the Pd^0^ nanoparticles on QuadraPure are analyzed using TEM (CM-12). The TEM micrograph and size distribution show that the Pd^0^ nanoparticles are well-dispersed onto the resin with an average size of 7.4 ± 3 nm in diameter (Figure 4). It is obviously shown that the nanoparticles are spherical in shape.

### 3.2. Suzuki Cross-Coupling Reactions

To investigate the catalytic activity of the XL-QPPd resin, Suzuki cross-coupling reactions are carried out in the presence of resin (5 mol% based on Pd content) in water for 10 mins under microwave condition (120°C, 100 w) under air condition (Table 1). The results shows that XL-QPPd resins are good catalysts for the Suzuki cross-coupling reaction by giving good yield with high purity for the aryl bromides and aryl iodides (Table 1, entry 1–8). However, the resins give poor yield with aryl chlorides under the same reaction condition (Table 1, Entries 9 and 10). Aryl chlorides are unreactive towards Suzuki cross-coupling reaction (relative reactivity: I > OTf > Br *⋙* Cl) due to the strength of the Ar–Cl bond (Ph-X: Cl (96) Br (81) > I (65 kcal/mol)) [30]. It hinders the formation of Ar–Cl bond between the organoboronic acid with aryl halides. After the completion of the reaction, the resin can be recovered by simple filtration and washed with solvents to remove all the impurities. Therefore, the XL-QPPd resins are good heterogeneous catalysts for Suzuki cross-coupling reaction (aryl bromide and aryl iodide).

A filtration test was carried out to investigate the amounts of active Pd species leach out into the solution during the reaction. Each supported Pd catalyst was placed under the Suzuki reaction condition (microwave, 120°C for 12 min) and supernatant collected. The filtrate was then used for a Suzuki cross-coupling reaction between 4-bromoacetophenone and boronic acid. The filtrate from XL-QPPd afforded conversions of 3%, indicating that minimal amount of palladium leached out from XL-QPPd.

**Figure 5:**
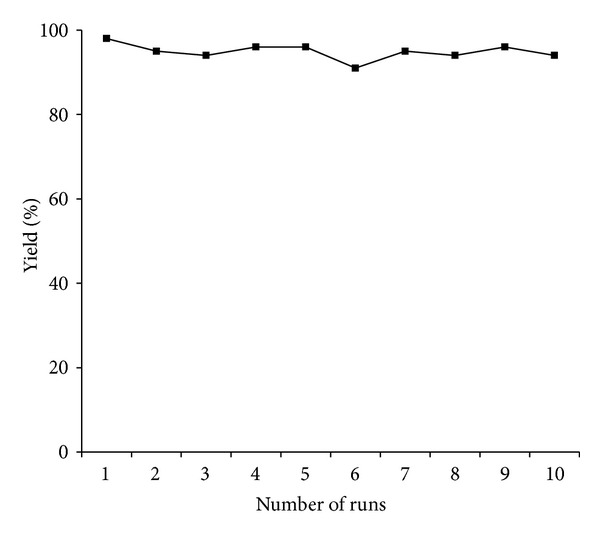
Recyclability of XL-QPPd resins on Suzuki cross-coupling reaction between the phenylboronic acid and 4-bromoacetophenone.

In order to investigate the recyclability of the XL-QPPd resins, the Suzuki cross-coupling reaction between the phenylboronic acid and 4-bromoacetophenone is examined (Figure 5). The catalysts are performed consistent catalytic activity after 10 runs. The results show that XL-QPPd resins can be reused for at least 10 times with no significant loss of catalytic activity, with simple filtration and washing. The leaching of Pd^0^ into the reaction mixture from the recovered XL-QPPd resins supported is analyzed using ICP-MS. The minimal amount of palladium leached out from XL-QPPd resins is only 0.21 ppm, which indicates that only small amount of catalysts leached out into the reaction mixture.

A comparison of the present method with others reported in the literature using nanoparticles and microwave heating was done and the results are summarized in Table 2. Palladium complex with salicylaldehyde N(4)-hexamethyleneiminylthiosemicarbazone was used as catalyst in Suzuki coupling reaction, but it took longer time to complete the reaction [31]. Chitosan-supported catalyst reported by Yi et al. showed higher yield in the presence of phase transfer agent TBAB in the reaction mixture [32]. In case of Tentagel and Macronet MN100 HPS (entries 3 and 4) needs extra cross-linking steps to reduce metal leaching problem [25, 33]. This better result can be attributed to a better solubilization of the base in a solvent with higher polarity. de Luna Martins et al. and Arvela and leadbeater have used Pd/C and Pd_2_dba_3_ for microwave-prompted Suzuki coupling reaction in toluene and DMF/H_2_O as solvent, but no recycling was reported [34, 35]. Qi and coworkers have reported that only 0.2 mol% loading of Pd (*γ*-Al_2_O_3_-Pd) was needed to obtain cross-coupling products in the presence of DMF/H_2_O. In the present work, a relatively high Pd loading was used, but it only needs a low amount of catalyst for the Suzuki reaction due to the high loading of QuadraPure resin.

## 4. Conclusions

In conclusion, we have successfully prepared the cross-linked captured palladium resin (XL-QPPd) as a heterogeneous catalyst via a simple method. The XL-QPPd exhibited a good catalytic activity for microwave-promoted Suzuki cross-coupling reactions under aerobic condition with low leaching of palladium from the resin. Moreover, the catalyst can be recovered and reused for several times without any loss of catalytic activity by simple filtration.

## Figures and Tables

**Table 1:** Microwave-promoted Suzuki coupling of aryl halides with phenylboronic acid catalyzed by XL-QPPd nanoparticles.

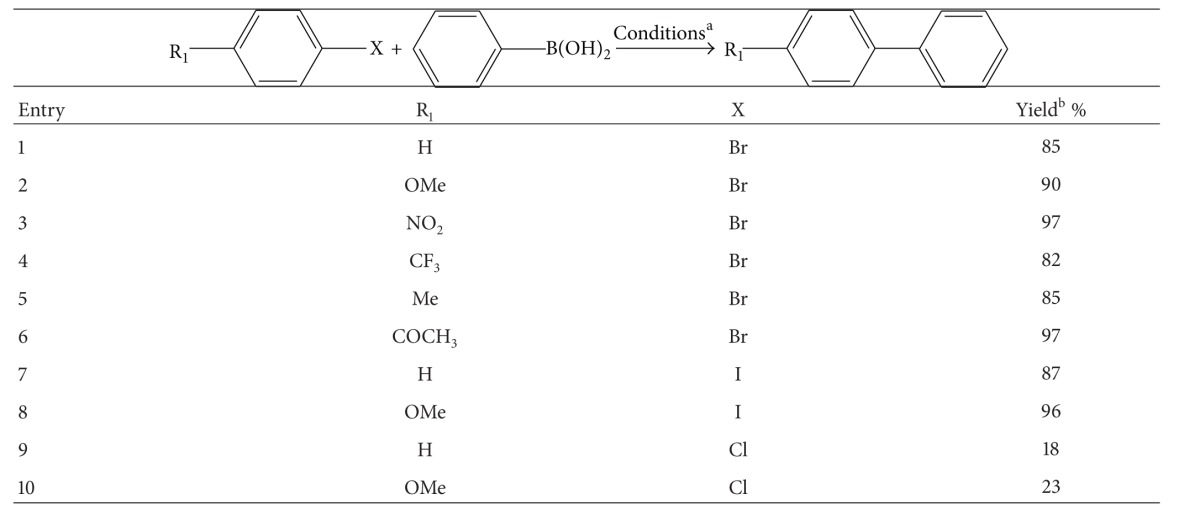

^a^Reaction condition: phenylboronic acid (0.75 mmol), aryl halide (0.5 mmol), K_2_CO_3_ (0.75 mmol), XL-QPPd (0.05 mmol), water (2 mL), and microwave, 120°C.

^b^Based on the isolated yield of the monosubstituted product after column chromatography on silica gel.

**Table 2:** Comparison with other catalyst systems.

Entry	Catalyst support	Pd (mol%)	Phase transfer agent/solvent	Reference
1	Pd complex	1 mM	DMF/H_2_O	[31]
2	Chitosan	0.5	TBAB/dioxane	[32]
3	Aminomethylated TentaGel	10.0	H_2_O	[25]
4	Macronet MN100 HPS	2.0	H_2_O	[33]
5	Pd_2_dba_3_	1.0	Toluene	[34]
6	Pd/C	1.0	DMF/H_2_O	[35]
7	*γ*-Al_2_O_3_-Pd	0.2	DMF/H_2_O	[36]
8	XL-QPPd	10.0	H_2_O	Present
